# Synthesis, HIV-1 RT inhibitory, antibacterial, antifungal and binding mode studies of some novel *N*-substituted 5-benzylidine-2,4-thiazolidinediones

**DOI:** 10.1186/s40199-014-0086-1

**Published:** 2015-01-24

**Authors:** Radhe Shyam Bahare, Swastika Ganguly, Kiattawee Choowongkomon, Supaporn Seetaha

**Affiliations:** Department of Pharmaceutical Sciences, Birla Institute of Technology, Mesra, Ranchi, 835215 Jharkhand India; Department of Biochemistry, Faculty of Science, Kasetsart University, Bangkean, Bangkok 10900 Thailand

**Keywords:** Antibacterial, Antifungal, Docking, HIV-1 RT inhibitory activity, Thiazolidinediones, Synthesis

## Abstract

**Background:**

Structural modifications of thiazolidinediones at 3^rd^ and 5^th^ position have exhibited significant biological activities. In view of the facts, and based on *in silico* studies carried out on thiazolidine-2,4-diones as HIV-1- RT inhibitors, a novel series of 2,4-thiazolidinedione analogs have been designed and synthesized.

**Methods:**

Title compounds were prepared by the reported method. Conformations of the structures were assigned on the basis of results of different spectral data. The assay of HIV-1 RT was done as reported by Silprasit *et al.* Antimicrobial activity was determined by two fold serial dilution method. Docking study was performed for the highest active compounds by using Glide 5.0.

**Results:**

The newly synthesized compounds were evaluated for their HIV-1 RT inhibitory activity. Among the synthesized compounds, compound **24** showed significant HIV-1 RT inhibitory activity with 73% of inhibition with an IC_50_ value of 1.31 μM. Compound **10** showed highest activity against all the bacterial strains.

A molecular modeling study was carried out in order to investigate the possible interactions of the highest active compounds **24**, **10** and **4** with the non nucleoside inhibitory binding pocket(NNIBP) of RT, active site of GlcN-6-P synthase and cytochrome P450 14-α-sterol demethylase from *Candida albicans* (*Candida* P450DM) as the target receptors respectively using the Extra Precision (XP) mode of Glide software.

**Conclusion:**

A series of novel substituted 2-(5-benzylidene-2,4-dioxothiazolidin-3-yl)-*N*-(phenyl)propanamides (**4**–**31**) have been synthesized and evaluated for their HIV-1 RT inhibitory activity, antibacterial and antifungal activities. Some of the compounds have shown significant activity. Molecular docking studies showed very good interaction.

## Background

The thiazolidinedione scaffold has been identified to play an essential role in medicinal chemistry [1,2]. Compounds containing the thiazolidinedione moiety have been found to exhibit a wide range of biological activities viz., antihyperglycemic [3], anti-inflammatory [4], antimalarial [5], antioxidant [6], antitumor [7], cytotoxic [8], antimicrobial [9], antiproliferative [10], MurD ligase inhibitor [11], monoamine oxidase B (MAO-B) inhibitor [12] neuroprotective [13], COX-2 inhibitor [14] and chemotherapeutic activities [15]. Recently, a novel series of thiazolidin-4-ones have emerged as selective NNRTIs [16]. However not much work has been reported on thiazolidine −2,4- diones as HIV-1-RT inhibitors.

HIV is the causative organism for AIDS and is continuously evolving and rapidly spreading throughout the world as a global infection. The HIV infection targets the monocytes expressing surface CD4 receptors and produces profound defects in cell-mediated immunity [17]. Overtime infection leads to severe depletion of CD4 T-lymphocytes (T-cells) resulting in opportunistic infections like tuberculosis (TB), fungal, viral, protozoal and neoplastic diseases and ultimately death [18].

Reverse transcription of the single-stranded (+) RNA genome into double-stranded DNA is an essential step in the HIV-1 replication life-cycle and requires the concerted function of both the DNA polymerase and ribonuclease H (RNase H) active sites of HIV-1 reverse transcriptase (RT). Due to its essential role in HIV-1 replication, RT is a major target for anti-HIV drug development and two classes of inhibitors, (1) the nucleoside and nucleotide RT inhibitors and (2) the nonnucleoside RT inhibitors (NNRTIs) have been approved by the United States Food and Drug Administration (FDA) for the treatment of HIV-1 infection [19]. Though the NNRTIs are effective and generally well-tolerated in the majority of patients, treatment durability is limited by drug-related side effects and rapid emergence of resistance among HIV isolates. Thus, the therapeutic efficacy of NNRTIs is mainly restricted due to development of viral resistance to NNRTIs associated with mutations that include K103N, L100I and Y188L, and with the development of second generation NNRTIs, the search for a more suitable NNRTI, which blocks the replication of all existing resistant viral strains and retains potency for longer periods of time by modifying the existing drug classes or by incorporating appropriate substitutions in the newer chemical scaffolds, according to the pharmacophoric requirements using multi-disciplinary approaches is the call of the day.

With the advent of AIDS and as a result of promiscuous use of drug therapy, antibacterial cytotoxins, steroids, or due to underlying disease or medical manipulation the normal defenses conferred by the microbial flora breaks down resulting in the prevalence of opportunistic bacterial and fungal infections. Bacterial diseases such as tuberculosis, typhus, plague, diphtheria, typhoid fever, cholera, dysentery and pneumonia have taken a high toll on humanity [20]. Along with this prevalence of multi-drug resistant microbial pathogens as an important and challenging therapeutic problem and therefore a search for newer antibacterial agents is the call of the day [21].

Opportunistic fungal infections have emerged as important causes of morbidity and mortality in immunocompromised patients and such infections include candidiasis, aspergillosis and mucormycosis [22]. A dramatic increase in invasive fungal infections over the past decade has been observed [23]. To overcome these problems, the development of new and safe antifungal agents with higher selectivity and lower toxicity is urgently required.

Glucosamine-6-phosphate synthase (GlcN-6-P synthase, L-glutamine:D-fructose-6P amidotransferase), is a new target for antibacterials [24] and antifungals [25]. GlcN-6-P synthase catalyzes the first step in hexosamine metabolism, converting fructose 6-phosphate into glucosamine 6-phosphate (GlcN6P) in the presence of glutamine. The reaction catalyzed by GlcN-6-P synthase is irreversible, and is therefore considered as a committed step. The end product of the pathway, N-acetyl glucosamine, is an essential building block of bacterial and fungal cell walls.

Recent modeling studies report that azoles may be acting as antimicrobials by inhibition of GlcN-6-P synthase [24].

The fungal cell wall, a structure essential to fungi and lacking in mammalian cells, is an obvious target for antifungal agents. Its major macromolecular components are chitin, ß-glucan, and mannoproteins [26]. In fungi, lanosterol 14-α-demethylase, a member of the cytochrome P450 superfamily, is an essential requirement for fungal viability. Azoles inhibit fungal cytochrome P-450 14-α-demethylase (DM) which is responsible for the conversion of lanosterol to ergosterol leading to the depletion of ergosterol in the fungal cell membrane [27–29]. Thus cytochrome P-450DM plays a key role in fungal sterol biosynthetic pathways, and this has been an important target for design of potent antifungals [30].

**Figure 1:**
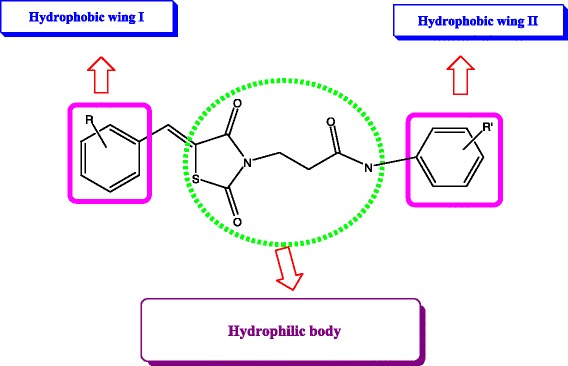
**Pharmacophoric model of 2,4-thiazolidinedione analogs.**

Structural modifications of thiazolidinediones at 3^rd^ and 5^th^ position have exhibited significant biological activities [31]. In view of the mentioned above facts, and based on *in silico* studies carried out on thiazolidine 2,4-diones as HIV-1- RT inhibitors [32], a novel series of 2,4-thiazolidinedione analogs have been designed based on the pharmacophoric model of NNRTIs 18 with the thiazolidinedione moiety attached to the propionamide moiety (−CH_2_-CH_2_-CO-NH-) constituting the “body (hydrophilic)” flanked by aryl rings (hydrophobic) linked to the 3rd and 5th position of the thiazolidinedione ring and to that of substituted aromatic amines as the “wings” to enhance the hydrophobicity of the molecules (Figure 1).

Herein we wish to report the synthesis of newer thiazolidine-2,4- diones, which have been evaluated for anti-HIV, antibacterial and antifungal activities. Binding mode analyses for the compounds with the highest HIV-1- RT inhibitory activity, antibacterial and antifungal activities have been carried out to understand the pharmacophoric features responsible for these activities.

## Experimental

### Materials

#### Synthetic studies

All reagents were purchased from commercial suppliers like Sigma Aldrich, Merck India Ltd., Himedia and Rankem chemicals. All reagents were GR or AR grade and were used without purification. The purity and homogeneity of the compounds were assessed by the TLC performed on Merck silica gel 60 F_254_ aluminium sheets using chloroform: methanol (9:1) as eluents. Iodine chamber and Shimadzu (UV-254) spectrometer were used for visualization of TLC spots. Ashless Whatmann No.1 filter paper was used for vacuum filtration. Melting points were determined on an SRS Opti-melting point automatic apparatus and were uncorrected. Elemental data of C, H and N were within ±0.4% of the theortical value as determined by Perkin Elmer Model 240 analyzer. IR spectra (KBr disc/or pallets) were recorded on SHIAMADZU FT/IR 8400 and were reported in cm.^−1^^1^ H-NMR and ^13^C NMR spectra were respectively recorded at 400 and 100 MHz with BRUKER Advance Digital Spectrophotometer. Chemical shifts are expressed in δ-values (ppm) relative to TMS as an internal standard, using DMSO-d_6._ Chemical shifts are expressed in δ-values (ppm) relative to TMS as an internal standard, using DMSO-d_6_ and Mass spectra were recorded with a AZILANT Q-TOF Micromass LC-MS by using (ESI+).

### Methods

#### General Procedure for the preparation of compounds (4–31)

Compounds **4**–**31** were synthesized as per the reported procedure [33]. Substituted 5-benzylidene-2,4-thiazolidinediones **(2a-l)** (0.01 mol) and the corresponding 3-chloro-N-phenylpropanamides **(3a-l)** (0.01 mol) were dissolved in 20 ml of acetonitrile. 0.02 mol of triethylamine was added dropwise to this solution with stirring. The reaction mixture was refluxed for 12 h, evaporated in rotary evaporator, cooled and poured into crushed ice and then basified with solid potassium carbonate. The resulting precipitate was filtered, washed with water (3 × 100 ml) and further washed with n-hexane (3 × 20 ml). The solid residue obtained was recrystallized from methanol to yield the desired compounds.

- **Thiazolidine-2,4-dione (1)**
  IR (KBr) cm^−1^: 3132 (NH stretching), 1741, 1681, 1586 (C = O), ^1^H-NMR (DMSO-d_6_, 400 MHz): 12.50 (s; 1H; NH), 4.39 (2H, s, CH_2_).
- **5-(benzylidene) thiazolidine-2,4-dione (2)**
  IR (KBr) cm^−1^: 3146 (NH stretching), 3039 (Ar-CH stretching), 2789 (C-CH stretching), 1741, 1693 (C = O stretching).
  ^1^H-NMR (DMSO-d_6_, 400 MHz): 9.94 (s; 1H; NH), 8.11 (s; 1H; C = CH), 8.09-6.91 (m, 5H, Ar-H).
- **2-chloro-N-phenylpropionamide (3)**
  IR (KBr) cm^−1^: 3138 (NH stretching), 1689 (C = O stretching), 1303 (C-CN stretching), ^1^H-NMR (DMSO-d_6_, 400 MHz): 8.60 (s; 1H; NH), 8.12-7.24 (5H, m, Ar-H), 4.82 (q; 1H; CH- CH_3_),1.58 (s; 3H; CH-CH_3_).
- **3-(5-benzylidene-2,4-dioxothiazolidin-3-yl)-*N*-(3-hydroxyphenyl)propanamide (4)**
  IR (KBr) cm^−1^: 3353 (OH stretching), 3153 (NH stretching), 1741,1676, 1648 (C = O stretching), 1329 (C-N aliphatic stretching), ^1^H-NMR (DMSO-d_6_, 400 MHz): 10.39 (s; 1H; 3′′OH), 9.83(s; 1H; NH), 7.80 (s;1H; C = CH), 7.51-6.85(m; 9H; Ar-H), 3.93 (t; 2H; N-CH_2_-CH_2_-CO), 2.66 (t; 2H; N-CH_2_-CH_2_-CO), ^13^C NMR(δ)DMSO-d_6_: 167.0, 166.0, 164.5 (C = O), 149.1 (C, Ar), 140.0 (C, Ar), 136.0 (=CH-), 135.1 (C, Ar), 130.0 (CH, Ar), 129.2 (2C, CH, Ar), 128.5 (2C, CH, Ar), 128.1, 124.3 (CH, Ar), 121.5 (C-5 TZD), 119.8 (CH, Ar), 117.1 (CH, Ar), 41.5 (CH_2_-CH_2_), 31.2 (CH_2_-CH_2_), MS (ESI+) m/z 369.0 (M+).
- **3-(5-(3-hydroxybenzylidene)-2,4-dioxothiazolidin-3-yl)-*N*-phenylpropanamide (5)**
  IR (KBr) cm^−1^: 3363 (OH stretching), 3153 (NH stretching), 3045, 2962 (Ar-CH stretching), 1676, 1648, 1640 (C = O stretching), ^1^H-NMR (DMSO-d_6_, 400 MHz): 10.02 (s; 1H; 3′OH), 9.84 (s; 1H; NH), 7.80 (s; 1H; C = CH), 7.51-6.85 (m; 9H; Ar-H), 3.93 (t; 2H; N-CH_2_-CH_2_-CO), 2.63 (t; 2H; N-CH_2_-CH_2_-CO), MS (ESI+) m/z 369.0 (M+).
- **3-(5-(2-fluorobenzylidene)-2,4-dioxothiazolidin-3-yl)-*N*-phenylpropanamide (6)**
  IR (KBr) cm^−1^: 3313 (NH stretching), 3051, 2929 (Ar-CH stretching), 1753, 1687, 1661 (C = O stretching), 1136 (C-F stretching), ^1^H-NMR (DMSO-d_6_, 400 MHz): 10.03 (s; 1H; NH), 7.88 (s; 1H; C = CH), 7.58-6.98 (m; 9H; Ar-H), 3.93 (t; 2H; N-CH_2_-CH_2_-CO), 2.66 (t; 2H; N-CH_2_-CH_2_-CO), MS (ESI+) m/z 371.0 (M+).
- **3-(5-(3-hydroxybenzylidene)-2,4-dioxothiazolidin-3-yl)-*N*-(3-hydroxyphenyl)propanamide (7)**
  IR(KBr) cm^−1^: 3525, 3416 (OH stretching), 3232 (NH stretching), 3053, 2947 (Ar-CH stretching),1722, 1664, 1652 (C = O stretching), ^1^H-NMR (DMSO-d_6_, 400 MHz): 10.00 (s;1H; NH), 9.79 (s; 1H; 3′OH), 9.78 (s; 1H; 3′′OH), 7.80 (s; 1H; C = CH), 7.51-6.85 (m; 8H; Ar-H), 3.90 (t; 2H; N-CH_2_-CH_2_-CO), 2.63 (t; 2H; N-CH_2_-CH_2_-CO), MS (ESI+) m/z 385.1 (M+).
- **3-(5-benzylidene-2,4-dioxothiazolidin-3-yl)-*N*-(2-chlorophenyl)propanamide (8)**
  IR (KBr) cm^−1^: 3306 (NH stretching), 1743, 1683, 1649 (C = O stretching), 821 (C-Cl stretching), ^1^H-NMR (DMSO-d_6_, 400 MHz): 10.02 (s; 1H; NH), 8.01(s; 1H; C = CH), 7.91 -7.32 (m; 9H; Ar-H), 3.93(t; 2H; N-CH_2_-CH_2_-CO), 2.63 (t; 2H; N-CH_2_-CH_2_-CO), MS (ESI+) m/z 386.0 (M+).
- **3-(5-(2,4-dimethylbenzylidene)-2,4-dioxothiazolidin-3-yl)-*N*-*p*-tolylpropanamide (9)**
  IR (KBr) cm^−1^: 3386 (NH stretching), 2935, 2852 (C-CH_3_ stretching), 1747, 1703, 1685 (C = O stretching), ^1^H-NMR (DMSO-d_6_, 400 MHz): 10.05 (s; 1H; NH), 8.06 (s; 1H; C = CH), 8.02-7.20 (m; 7H; Ar-H), 3.33 (t; 2H; N-CH_2_-CH_2_-CO), 2.66 (t; 2H; N-CH_2_-CH_2_-CO), 2.30 (s; 6H; 4′,4′′CH_3_), 2.24 (s; 3H; 4′′CH_3_), MS (ESI+) m/z 395.2 (M+).
- **3-(5-(2,5-dimethylbenzylidene)-2,4-dioxothiazolidin-3-yl)*-N*-(2-hydroxyphenyl)propanamide (10)**
  IR (KBr) cm^−1^: 3389 (OH stretching), 3198 (NH stretching), 2995, 2885 (C-CH_3_ stretching) 1668, 1646, 1640 (C = O stretching),^1^H-NMR (DMSO-d_6_, 400 MHz): 10.20 (s; 1H; OH), 10.11 (s; 1H; NH), 7.90 (s; 1H; C = CH), 7.61-7.29 (m; 7H; Ar-H), 3.93 (t; 2H; N-CH_2_-CH_2_-CO), 2.70 (t; 2H; CH_2_), 2.45 (s; 3H; 2′CH_3_), 2.24 (s; 3H; 5′CH_3_), ^13^C NMR(δ)DMSO-d_6_: 166.7, 166.4, 164.5 (C = O), 148.1 (C, Ar), 135.8, 134.7, 133.0 (C, Ar), 132.8 (=CH-), 130.2, 129.0, 127.2, 125.4 (CH, Ar), 122.2 (CH, Ar), 121.5 (C-5 TZD), 120.2, 119.2 (CH, Ar), 116.0 (CH, Ar), 121.0, 119.4 (C-CH_3_), 41.5 (CH_2_-CH_2_), 31.2 (CH_2_-CH_2_), MS (ESI+) m/z 397.2 (M+).
- **3-(5-(2,4-dimethylbenzylidene)-2,4-dioxothiazolidin-3-yl)-*N*-(2-hydroxyphenyl)propanamide (11)**
  IR (KBr) cm^−1^: 3404 (OH stretching), 3136 (NH stretching), 2742 (C-CH_3_ stretching), 1734, 1681, 1641 (C = O stretching), 665 (C-S stretching), ^1^H-NMR (DMSO-d_6_, 400 MHz): 10.15 (s; 1H; 2′OH), 10.03 (s; 1H; NH), 8.06 (s; 1H; C = CH), 8.03 -7.20 (m; 7H; Ar-H), 3.92 (t; 2H; N-CH_2_-CH_2_-CO), 2.66 (s; tH; CH_2_), 2.29 (s; 3H; 2′CH_3_), 2.23 (s; 3H; 4′CH_3_), MS (ESI+) m/z 397.2 (M+).
- **3-(5-(3,5-dimethylbenzylidene)-2,4-dioxothiazolidin-3-yl)-*N*-(2-hydroxyphenyl)propanamide (12)**
  IR (KBr) cm^−1^: 3304 (OH stretching), 3203 (NH stretching), 2980 (C-CH_3_ stretching), 1658, 1650, 1642 (C = O stretching), 690 (C-S stretching), ^1^H-NMR (DMSO-d_6_, 400 MHz): 10.20 (s; 1H; 2′′OH), 10.11 (s; 1H; NH), 7.90 (s; 1H; C = CH), 7.61-7.27 (m; 7H; Ar-H), 3.90 (t; 2H; N-CH_2_-CH_2_-CO), 2.63 (s; tH; CH_2_), 2.24-2.27 (s; 6H; 2′5′CH_3_), MS (ESI+) m/z 397.0 (M+).
- **3-(5-(2,4-dihydroxybenzylidene)-2,4-dioxothiazolidin-3-yl)-*N*-(2-hydroxyphenyl)propanamide (13)**
  IR (KBr) cm^−1^: 3657, 3566, 3412 (OH stretching), 3390 (NH stretching), 3087, 3032 (Ar-CH stretching), 1687, 1669, 1656 (C = O stretching), ^1^H-NMR (DMSO-d_6_, 400 MHz): 12.44 (s; 1H; 2′′OH), 10.70 (s; 1H; 2′OH), 10.01 (s; 1H; 4′OH), 9.98 (s; 1H; NH), 8.01 (s; 1H; C = CH), 8.03-7.24 (m; 7H; Ar-H), 3.98 (t; 2H; N-CH_2_-CH_2_-CO), 2.23 (t; 2H; 2 CH_2_), MS (ESI+) m/z 401.2 (M+).
- **3-(5-benzylidene-2,4-dioxothiazolidin-3-yl)-*N*-(2-chloro-4-methylphenyl)propanamide (14)**
  IR (KBr) cm^−1^: 3281 (NH stretching), 1664, 1650 (C = O stretching), 1329 (C-N aromatic stretching), 783 (C-Cl stretching), ^1^H-NMR (DMSO-d_6_, 400 MHz): 10.17 (s; 1H; NH), 7.93 (s; 1H; C = CH), 7.64 -6.44 (m; 8H; Ar-H), 2.66-3.96 (t; 2H; N-CH_2_-CH_2_-CO), 2.31 (t; 2H; N-CH_2_-CH_2_-CO), 2.26 (s; 3H; 4′′CH_3_), MS (ESI+) m/z 401.5 (M+).
- **3-(5-benzylidene-2,4-dioxothiazolidin-3-yl)-*N*-(4-chloro-3-methylphenyl)propanamide (15)**
  IR (KBr) cm^−1^: 3283 (NH stretching), 1684, 1654, 1609 (C = O stretching), 1305 (C-N aromatic stretching), 761 (C-Cl stretching), ^1^H-NMR (DMSO-d_6_, 400 MHz): 10.19 (s; 1H; NH), 7.93 (s; 1H; C = CH), 7.93-6.23 (m; 8H; Ar-H), 2.67-3.95 (t; 2H; N-CH_2_-CH_2_-CO), 2.30 (s; 3H; 4′′CH_3_), 2.27 (t; 2H; N-CH_2_-CH_2_-CO), MS (ESI+) m/z 401.3 (M+).
- **3-(5-(2-chlorobenzylidene)-2,4-dioxothiazolidin-3-yl)-*N*-*p*-tolylpropanamide (16)**
  IR (KBr) cm^−1^: 3215 (NH stretching) 1666, 1655, 1645 (C = O stretching), 717 (C-Cl stretching),^1^H-NMR (DMSO-d_6_, 400 MHz): 10.02 (s; 1H; NH), 7.88 (s; 1H; C = CH), 7.58 -7.23 (m; 8H; Ar-H), 3.94 (t; 2H; N-CH_2_-CH_2_-CO), 2.67 (t; 2H; N-CH_2_-CH_2_-CO), 2.30 (s; 3H; 4′′CH_3_), MS (ESI+) m/z 401.3 (M+).
- **3-(5-(2,3,4-trihydroxybenzylidene)2,4-dioxothiazolidin-3-yl)-*N*-*p*-tolylpropanamide (17)**
  IR(KBr)cm^−1^: 3649, 3629, 3595 (OH-stretching), 3312 (NH-stretching), 2869 (C-CH_3_ stretching), 1745, 1683,1656 (C = O stretching), ^1^H-NMR (DMSO-d_6_, 400 MHz): 14.09 (s; 1H; 2′OH), 10.09 (s; 1H; 3′OH), 9.50 (s; 1H; 4′OH), 9.31 (s;1H; NH), 8.06(s;1H; C = CH), 8.04 -7.20 (m; 6H; Ar-H), 3.84 (t; 2H; N-CH_2_-CH_2_-CO), 2.39 (s; 3H; 4′′CH_3_), 2.33 (t; 2H; N-CH_2_-CH_2_-CO), MS (ESI+) m/z 415.3 (M+).
- **3-(5-(3-hydroxybenzylidene)-2,4-dioxothiazolidin-3-yl)-*N*-(2-chloro-5-methylphenyl)- propanamide (18)**
  IR (KBr) cm^−1^: 3308 (OH stretching), 3223 (NH stretching), 3057, 2960 (Ar-CH stretching), 2924, 2854 (C-CH_3_ stretching), 1691,1656, 1646 (C = O stretching), 752 (C-Cl stretching), ^1^H-NMR (DMSO-d_6_, 400 MHz): 10.19 (s; 1H; 3′OH), 10.11 (s; 1H; NH), 7.93 (s; 1H; C = CH), 7.64-6.23 (m; 7H; Ar-H), 3.96-2.66 (t; 2H; N-CH_2_-CH_2_-CO), 2.30 (s; 3H; 5′′CH_3_), 2.27 (t; 2H; N-CH_2_-CH_2_-CO), MS (ESI+) m/z 417.2 (M+).
- **3-(5-(2-fluorobenzylidene)-2,4-dioxothiazolidin-3-yl)-*N*-(2-chloro-4-methylphenyl)- propanamide (19)**
  IR (KBr) cm^−1^: 3306 (NH stretching), 2926, 2856 (C-CH_3_ stretching), 1743, 1693, 1654 (C = O stretching), 1282 (C-F stretching), 754 (C-Cl stretching), ^1^H-NMR (DMSO-d_6_, 400 MHz): 10.02 (s; 1H; NH), 7.88 (s; 1H; C = CH), 7.58-7.23 (m; 7H; Ar-H), 3.99 ((t; 2H; N-CH_2_-CH_2_-CO), 2.60 (t; 2H; N-CH_2_-CH_2_-CO), 2.28 (s; 3H; 4′′CH_3_), MS (ESI+) m/z 419.2 (M+).
- **3-(5-(3,5-dimethylbenzylidene)-2,4-dioxothiazolidin-3-yl)-*N*-(2-chloro-4-methylphenyl)- propanamide (20)**
  IR (KBr) cm^−1^: 3267 (NH stretching), 3039 (Ar-CH stretching), 2916, 2858 (C-CH_3_ stretching), 1681, 1651,1644 (C = O stretching), 742 (C-Cl stretching) ^1^H-NMR (DMSO-d_6_, 400 MHz): 10.07 (s; 1H; NH), 8.04 (s; 1H; C = CH), 7.84-7.20 (m; 6H; Ar-H), 3.92 (t; 2H; N-CH_2_-CH_2_-CO), 2.66 (t; 2H; N-CH_2_-CH_2_-CO), 2.30 (s; 6H; CH_3_), 2.24 (s; 3H; 4’CH_3_).
- **3-(5-(2-chlorobenzylidene)-2,4-dioxothiazolidin-3-yl)-*N*-(4-nitrophenyl)propanamide (21)**
  IR (KBr) cm^−1^: 3145 (NH stretching), 1685, 1657, 1644 (C = O stretching), 1311 (C-NO_2_ stretching), 776 (C-Cl stretching),^1^H-NMR (DMSO-d_6_, 400 MHz): 10.39 (s; 1H; NH), 8.01(s;1H; =CH), 7.91-7.32 (m; 8H; Ar-H), 3.91 (t; 2H; N-CH_2_-CH_2_-CO), 2.72 (t; 2H; N-CH_2_-CH_2_-CO), MS (ESI+) m/z 432.8 (M+).
- **3-(5-(2-chlorobenzylidene)-2,4-dioxothiazolidin-3-yl)-*N*-(2-nitrophenyl)propanamide (22)**
  IR (KBr) cm^−1^: 3306 (NH stretching), 1743, 1693, 1612 (C = O stretching), 1342 (C-NO_2_ stretching), 754 (C-Cl stretching), ^1^H-NMR (DMSO-d_6_, 400 MHz): 10.00 (s; 1H; NH), 7.80 (s; 1H;C = CH), 7.51-6.85 (m; 8H; Ar-H), 3.90 (t; 2H; N-CH_2_-CH_2_-CO), 2.63 (t; 2H; N-CH_2_-CH_2_-CO).
- **3-(5-(4-chlorobenzylidene)-2,4-dioxothiazolidin-3-yl)-*N*-(2-nitrophenyl)propanamide (23)**
  IR (KBr) cm^−1^: 3267 (NH stretching), 1702, 1664, 1650 (C = O stretching), 1373 (C-NO_2_ stretching), 752 (C-Cl stretching), ^1^H-NMR (DMSO-d_6_, 400 MHz): 10.00 (s; 1H; NH), 7.80 (s; 1H; C = CH), 7.51 -6.87 (m; 8H; Ar-H), 3.90 (t; 2H; N-CH_2_-CH_2_-CO), 2.63 (t; 2H; N-CH_2_-CH_2_-CO).
- **3-(5-(2,3,4-trihydroxybenzylidene)-2,4-dioxothiazolidin-3-yl)-*N*-(2-mercaptophenyl) propanamide (24)**
  IR (KBr) cm^−1^: 3649, 3629, 3587(OH stretching), 3312(NH stretching) 2975, 2931 (Ar-CH stretching), 2896 (C-CH_3_ stretching), 2546 (SH-stretching), 1688, 1647, 1638 (C = O stretching), ^1^H-NMR (DMSO-d_6_, 400 MHz): 13.96 (s; 1H; 2′OH), 10.09 (s; 1H; 3′OH), 9.50 (s; 1H; 4′OH), 9.31 (s;1H; NH), 8.01 (s;1H; C = CH), 7.90-7.18 (m; 6H; Ar-H), 3.84 (t; 2H; N-CH_2_-CH_2_-CO), 3.49 (s; 1H; 2′′SH), 2.30 (t; 2H; N-CH_2_-CH_2_-CO), ^13^CNMR(δ)DMSO-d_6_: 167.2, 165.5, 164.5 (C = O), 148.2, 145.2, 142.1, 136.0 (C, Ar), 136.1 (=CH-), 130.2, 129.0, 125.8 (CH, Ar), 124.3 (C, Ar), 123.6, 123.0 (CH, Ar), 121.5 (C-5 TZD), 110.1, (C, Ar), 109.3 (CH, Ar), 41.5 (CH_2_-CH_2_), 31.2 (CH_2_-CH_2_), MS (ESI+) m/z 432.8 (M+).
- **3-(5-(2,4-dihydroxybenzylidene)-2,4-dioxothiazolidin-3-yl)-*N*-(3-chloro-2-methylphenyl)propanamide (25)**
  IR (KBr) cm^−1^: 3630, 3444 (OH stretching), 3273 (NH stretching), 3053(Ar-CH stretching), 2793 (C-CH_3_ stretching), 1730, 1674, 1648 (C = O stretching), 792 (C-Cl stretching), ^1^H-NMR (DMSO-d_6_, 400 MHz): 10.51 (s; 1H; 2′OH), 10.19 (s; 1H; 4′OH), 9.69 (s; 1H; NH), 8.09 (s; 1H; C = CH), 7.41-7.16 (m; 6H; Ar-H), 3.95 (t; 2H; N-CH_2_-CH_2_-CO), 2.68 (t; 2H; N-CH_2_-CH_2_-CO), 2.19 (s; 3H; 2′′CH_3_), MS (ESI+) m/z 432.9 (M+).
- **3-(5-(4-chlorobenzylidene)-2,4-dioxothiazolidin-3-yl)-*N*-(2-chloro-4-methylphenyl)propanamide (26)**
  IR (KBr) cm^−1^: 3198 (NH stretching), 2918, 2848 (C-CH_3_ stretching), 1750, 1672, 1658 (C = O stretching), 1307 (C-N aromatic stretching), 748 (C-Cl stretching), ^1^H-NMR (DMSO-d_6_, 400 MHz): 9.65 (s; 1H; NH), 7.94 (s; 1H; C = CH), 7.90 -6.53 (m; 7H; Ar-H), 3.94-3.91 (t; 2H; N-CH_2_-CH_2_-CO), 2.70-2.76 (t; 2H; N-CH_2_-CH_2_-CO), 2.32 (s; 3H; CH_3_), ^13^C NMR(δ)DMSO-d_6_: 167.6, 165.8, 164.5 (C = O), 135.7 (=CH-), 135.6, 133.0, 131.9, 132.0, 131.0 (C, Ar), 130.0 (3C, CH, Ar) 127.8, (3C, CH, Ar), 124.2 (CH, Ar), 121.5 (C-5 TZD), 113.1, (C, Ar), 112.2, 109.5, 103.7 (CH, Ar), 41.5 (CH_2_-CH_2_), 31.2 (CH_2_-CH_2_), 20.7 (CH_3_), MS (ESI+) m/z 436.2 (M+).
- **3-(5-(4-chlorobenzylidene)-2,4-dioxothiazolidin-3-yl)-*N*-(2-chloro-5-methylphenyl)propanamide (27)**
  IR (KBr) cm^−1^: 3273 (NH stretching), 1739, 1670, 1652 (C = O stretching), 808 (C-Cl stretching), ^1^H-NMR (DMSO-d_6_, 400 MHz): 9.64 (s; 1H; NH), 7.95 (s; 1H; C = CH), 7.62 -6.20 (m; 7H; Ar-H), 3.94-3.20 (t; 2H; N-CH_2_-CH_2_-CO), 2.76-2.32 (t; 2H; N-CH_2_-CH_2_-CO), 2.23 (s; 3H; 5′′CH_3_), MS (ESI+) m/z 435.9 (M+).
- **3-(5-(2-bromobenzylidene)-2,4-dioxothiazolidin-3-yl)-*N*-(2-hydroxyphenyl)propanamide (28)**
  IR (KBr) cm^−1^: 3411 (OH stretching), 3372 (NH stretching), 1688, 1669, 1646 (C = O stretching), 668 (C-Br stretching), ^1^H-NMR (DMSO-d_6_, 400 MHz): 9.97 (s; 1H; 2′′OH), 9.84 (s; 1H; NH), 7.80 (s; 1H; C = CH), 7.51-6.85 (m; 8H; Ar-H), 3.91 (t; 2H; N-CH_2_-CH_2_-CO), 2.64 (t; 2H; N-CH_2_-CH_2_-CO), ^13^CNMR(δ)DMSO-d_6_: 167.2, 165.2, 164.5 (C = O), 148.9, 138.1, 135.3 (=CH-), 135.1 (C, Ar), 132.3, 130.1, 128.1, 127.1, 127.0 (CH, Ar), 121.5 (C-5 TZD), 113.0, (C, Ar), 112.9, 109.5, 104.8 (CH, Ar), 41.5 (CH_2_-CH_2_), 31.2 (CH_2_-CH_2_), MS (ESI+) m/z 448.0.
- **3-(5-(2-bromobenzylidene)-2,4-dioxothiazolidin-3-yl)-*N*-(3-hydroxyphenyl)propanamide (29)**
  IR (KBr) cm^−1^: 3444 (OH stretching), 3315 (NH stretching), 1752, 1680, 1650 (C = O stretching), 680 (C-Br stretching), ^1^H-NMR (DMSO-d_6_, 400 MHz): 9.84 (s; 1H; OH), 9.61 (s; 1H; NH), 7.81 (s; 1H; C = CH), 7.52-6.86 (m; 8H; Ar-H), 3.91 (t; 2H; N-CH_2_-CH_2_-CO), 2.64 (t; 2H; N-CH_2_-CH_2_-CO), MS (ESI+) m/z 448.2 (M+).
- **3-(5-(2-bromobenzylidene)-2,4-dioxothiazolidin-3-yl)-*N*-(4-nitrophenyl)propanamide (30)**
  IR (KBr) cm^−1^: 3207 (NH stretching), 1710, 1685, 1658 (C = O stretching), 1319 (C-NO_2_ stretching), 750 (C-Br stretching), ^1^H-NMR (DMSO-d_6_, 400 MHz): 9.73 (s; 1H; NH), 8.12 (s; 1H; C = CH), 8.09 -7.53 (m; 8H; Ar-H), 3.83 ((t; 2H; N-CH_2_-CH_2_-CO), 2.20 (t; 2H; N-CH_2_-CH_2_-CO).
- **3-(5-(3-bromobenzylidene)-2,4-dioxothiazolidin-3-yl)-*N*-(4-chloro-3-methylphenyl) propanamide (31)**
  IR (KBr) cm^−1^: 3284 (NH stretching), 3014, 2922 (C-CH_3_ stretching) 1702, 1676, 1652 (C = O stretching), 779 (C-Br Stretching), 675 (C-Br stretching), ^1^H-NMR (DMSO-d_6_, 400 MHz): 10.11 (s; 1H; NH), 7.90 (s; 1H; C = CH), 7.61 -7.29 (m; 7H; Ar-H), 3.90 (t; 2H; N-CH_2_-CH_2_-CO), 2.64 (t; 2H; N-CH_2_-CH_2_-CO), 2.45 (s; 3H; 3′′CH_3_), MS (ESI+) m/z 480.2 (M+).

## Biological assays

The standard strains were procured from Institute of Microbial Technology, Chandigarh and National Chemical Laboratory, Pune. Antimicrobial activity was determined by two fold serial dilution method [34] in duplicates against pathogenic microorganisms Gram-positive bacteria: *Staphylococcus aureus* (NCIM 2122), *Bacillus subtilis* (MTCC 121), Gram-negative bacteria: *Escherichia coli* (MTCC118), *Pseudomonas aeruginosa* (MTCC 647), *Salmonella typhi* (NCIM 2501), *Klebsiella pneumonia *(MTCC 3384) and fungus *Candida albicans* (MTCC 227), *Aspergillus niger* (NCIM 1056). Test compounds were dissolved in 10% DMSO, to produce a 2000 μg/ml stock solution. These test tubes were serially diluted to give a concentration of 100, 50, 25, 12.5, 6.25, 3.125, 1.56, and 0.78 μg/mL. MHB (Mueller-Hinton Broth) was used for bacteria and SDB (Sabouraud Dextrose Broth) was used for fungus. The cell density of each inoculum was adjusted in sterile water of a 0.5 McFarland standard. A final concentration of ~10^7^ CFU/mL and ~10^6^ CFU/mL was obtained for bacteria and fungus, respectively. Microbial inocula were added to the twofold diluted samples. The test tubes were incubated 18–24 h at 37° C ±1°C for bacteria and 2–5 days at 25°C ±1°C for fungus. Ciprofloxacin and fluconazole were used as standard drugs. The highest dilution of the test compound that completely inhibited the growth of test organism was considered as the MIC value of the test compound and was expressed in μg/ml.

### HIV-1 reverse transcriptase inhibition assay

The assay of HIV-1 RT was done as reported by Silprasit *et al.* [35]. All reagents used were provided in the EnzChek® Reverse Transcriptase Assay Kit (Molecular Probes, USA). A mixture of 5 μL of 1 mg/mL 350 bases-poly(rA) ribonucleotide template and 5 μL of 50 μg/mL oligo d(T)_16_ primer in a nuclease-free microcentrifuge tube were incubated at room temperature (25°C) for 1 hour to allow the primer/template annealing. The primer/template was prepared by 200-fold dilution in polymerization buffer. The primer/template was aliquoted and kept at −20°C until used. Five microliter of 8 μM stock purified HIV-1 reverse transcriptase was aliquoted and kept at −80°C until used. The working enzyme was diluted to 400 nM with 50 mM Tris–HCl, 20% glycerol, 2 mM DTT, pH 7.5.

The assays were performed in a total volume 15 μL of the polymerization reaction. The reaction containing 3 μl of 400 nM recombinant HIV-1 RT (the final concentration is 80 nM), 2 μL of TE buffer (10 mM Tris–HCl at pH 7.5) were added and gently mixed on ice prior. The polymerization reaction was initiated by the addition of 10 μL of the primer/template and incubated at room temperature for 30 minutes. After the reactions reached the desired incubation time, 5 μL of 0.2 M EDTA was added to stop the polymerization reaction (RTControl). The blank reaction was prepared by mixing 5 μL of 0.2 M EDTA with enzyme before adding primer/template (RTBlank). After termination of the reactions, the plate was gently shaken and incubated at room temperature for 3 min to allow the formation of a stable heteroduplex DNA/RNA complex, followed by addition of 180 μL of PicoGreen reagent diluted 700-fold with TE buffer for each well, making the final volume 200 μL and incubation at room temperature in the dark for 3 min, during which PicoGreen binds to double-stranded DNA and RNA-DNA hybrids, was followed by measurement of fluorescence with a fluorometer (excitation 485 nm; emission 535 nm).

To test the inhibition efficiency of the compounds, all the compounds were dissolved in dimethyl sulfoxide (DMSO) to make a 20 mM stock solution. Ten micromolar working solution of each inhibitor was further diluted by 10 mM Tris–HCl, pH 7.5 containing 50% DMSO. Two microliter of each inhibitor and 3 μL of 400 nM recombinant HIV-1 RT were added and gently mixed on ice prior. The reaction was initiated by the addition of 10 μL of the primer/template and incubated at room temperature for 30 minutes. After the reactions reached the desired incubation time, 5 μL of 0.2 M EDTA was added to stop the polymerization reaction. The relative inhibitory effect of HIV-1 RT activity was compared by using percent inhibition, which was calculated via the following eq (1):

1 $$ \%\ \mathrm{relative}\ \mathrm{inhibition}=\frac{\left[\left({\mathrm{RT}}_{\mathrm{Control}} - {\mathrm{RT}}_{\mathrm{Background}}\right) - \left({\mathrm{RT}}_{\mathrm{Sample}} - {\mathrm{RT}}_{\mathrm{Background}}\right)\right]}{\left[\left({\mathrm{RT}}_{\mathrm{Control}} - {\mathrm{RT}}_{\mathrm{Background}}\right)\right]}\times 100 $$

### Determination of the IC_50_ inhibition value

Determination of the IC_50_ was done by adding 2 μL of each two-fold serial dilution of inhibitors. Two microliters of each test compound was diluted serially 2-fold. Then, 2 μL of 30 ng/μL purified HIV-1 RT was added and mixed. A volume of 4 μL of the template/primer polymerization buffer was added into each well. The mixtures were incubated at 37°C for 10 min. The reactions were stopped with 5 μL of 200 mM EDTA and immediately incubated on ice for 30 min. The activity was determined by the PicoGreen–fluorometric method. The reaction was repeated three times and were determined using Graph pad Prism4 version with a non-linear regression model.

## Computational method with Glide 5.0

Docking study was performed for the highest active compounds by using Glide 5.0 (Schrodinger) [36] installed in a single machine running on a 3.4 GHz Pentium 4 processor with 1GB RAM and 160 GB Hard Disk with Red Hat Linux Enterprise version 5.0 as the operating system.

### Protein structure preparation

The X-ray crystallographic structure of (PDB code 1RT2, 2VF5 and 1EA1) was obtained from Brookhaven Protein Data Bank (RCSB) [37]. All water molecules were removed from the complex, and the protein was minimized using the protein preparation wizard. Partial atomic charges were assigned according to the OPLS_AA force field. A radius of 10 Å was selected for active site cavity during receptor grid generation for 2VF5. After assigning charge and protonation state finally refinement (energy minimization) was done using MM3 force field runs.

Crystallographic structure of the complex between cytochrome P450 14-R-sterol demethylase from *Mycobacterium tuberculosis* (*Mycobacterium* P450DM) and fluconazole was present in the Protein Data Bank with the ID 1EA1 [38]. The high homology existing between these two analogous enzymes [39] suggested building a simple model consisting of the crystallographic structure of the complex 1EA1 in which the residues that are arranged in a range of 7 Å from fluconazole, were substituted with those of *Candida* P450DM according to reported method [33]. Only 12 substitutions were made by replacement of the residues Pro77, Phe78, Met79, Arg96, Met99, Leu100, Phe255, Ala256, His258, Ile322, Ile323 and Leu324 by Lys77, His78, Leu79, Leu96, Lys99, Phe100, Met255, Gly256, Gln258, His322, Ser323 and Ile324, which were thought to be necessary for the ligand-receptor interaction. The complex between the chimeric enzyme thus obtained and was then minimized.

### Ligand structure preparation

All the compounds used in the docking study with Glide were built within maestro by using build module of Schrodinger Suite 2008. These structures were geometry optimized by using the Optimized Potentials for Liquid Simulations-2005 (OPLS_2005) force field with the steepest descent protocol followed by truncated Newton conjugate gradient protocol. Partial atomic charges were computed using the OPLS_2005 force field.

### Docking protocol and their validation

All docking calculations were then performed using the “Extra Precision” (XP) mode of Glide Program 5.0. A grid was prepared with the center defined by the co-crystallized ligand. During the docking process, initially Glide performed a complete systematic search of the conformational, orientational and positional space of the docked ligand and eliminated unwanted conformations using scoring followed by energy optimization. Finally the conformations were further refined via Monte Carlo sampling of pose conformation. Predicting the binding affinity and rank-ordering ligands in database screens was implemented by modified and expanded version of the Glide scoring function. The most suitable method of evaluating the accuracy of a docking procedure is to determine how closely the lowest energy pose predicted by the scoring function resembles an experimental binding mode as determined by X-ray crystallography. Docking validation was performed with an obtained RMSD value of 0.370 Å for 1RT2, 1.674 Å for 2VF5 and 2.094 Å for 1EA1 ensuring precision and reproducibility of the docking process.

## Results and discussion

### Chemistry

**Scheme 1:**
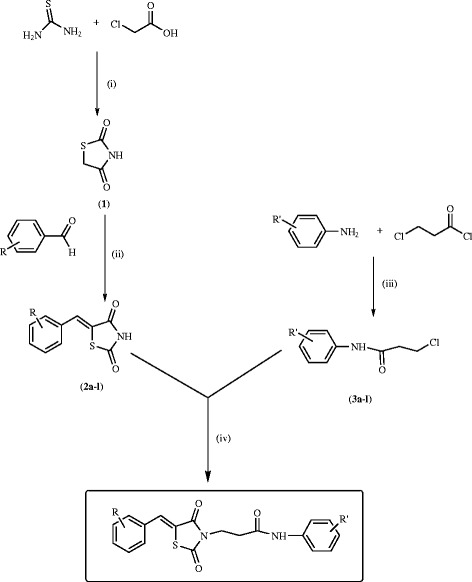
**Reagents and conditions: (i) Water, Conc. HCl, reflux 10-12 h (ii) ethanol, piperidine, reflu 4 h (iii) Glacial acetic acid (GAA), 0-5°C, 0.5 h, 4rt, stirring (iv) CH3CN, triethylamine, reflux 12h.** General scheme for the synthesis of compounds (4–31).

An attempt has been made to incorporate aryl groups in the 3^rd^ and 5^th^ position of the thiazolidinedione structure according to Scheme 1. In the first step the cyclization of chloroacetic acid was carried out with equilmolar amounts of thiourea and chloroacetic acid, and then hydrolysed with 2 N HCl to afford 2,4-thiazolidinedione (**1**). Knoevenagel condensation of 2,4-thiazolidinedione and appropriate aryl aldehydes was carried out in ethanol under reflux conditions containing catalytic amount of piperidine, as base, to form the corresponding substituted 5-benzylidene-2,4-thiazolidinediones (**2**) [33]. *N*-chloro-3-(phenylamino)propanamides (**3**) were prepared by the reported method [40]. Substituted *N*-chloro-3-(phenylamino)propanamides (**3**) were thus prepared by reacting appropriate aryl amines with 3-chloropropionyl chloride in the presence of glacial acetic acid in cold condition. The substituted 5-benzylidene-2,4-thiazolidinediones (**2**) were condensed with substituted *N*-chloro-3-(phenylamino)propanamides (**3**) in the presence of triethylamine using acetonitrile as the solvent to get substituted 3-(5-benzylidene-2,4-dioxothiazolidin-3-yl)-*N*-phenylpropanamides ***4****–****31***.

The physical data are given in Table 1. FTIR, ^1^H-NMR and mass spectral data for all the synthesized compounds are given in the above experimental protocols. The IR spectrum of all the final compounds exhibited very similar features and showed the expected bands for the characteristic groups present in the compounds such as N-H stretching in the range of 3390–3136 cm^−1^ and C = O stretching in the range of 1750–1645 cm^−1^ to confirm the presence of thiazolidinedione ring system. ^1^H NMR showed a characteristic singlet peak assigned to a δ value in the range of 10.39-9.31 thus confirming the presence of NH proton of thiazolidinedione scaffold in compounds **4**–**31**. The methylenic (C = CH) protons of compounds **4**–**31** were seen as a singlet between 8.12-7.80 δppm while aromatic protons appeared as multiple peaks within the range 8.04-6.20 δ ppm. Characteristic triplet of 2 protons was assigned at δ ranging 3.99-2.66 to the methylenic protons of N-CH_2_-CH_2_-CO. Similarly; a triplet of 2 protons was assigned to the methylenic protons of N-CH_2_-CH_2_-CO observed at δ ranging 2.76-2.20 for all the compounds **4**–**31**. The ^13^C NMR depicted the peaks of thiazolidine-2,4-dione (TZD) nucleus within the range δ 166.2-164.3 (thiazolidine-2,4-dione -C = O). ^13^C NMR spectrum showed signals for thiazolidine-2,4-dione-C-5 atom at δ 121.5.

**Table 1:** **Physical data of synthesized compounds (4–31)**

**Comp. no.**	**R**	**R'**	**Mol. formula**	**Yield (%)**	**M.P. (°C)**	**M.W.**	**Elemental analysis calculated/found**		
							**C**	**H**	**N**
4	H	3-OH	C_19_H_16_N_2_O_4_S	57	289	368.41	61.94	4.38	7.60
							61.90	4.34	7.55
5	3-OH	H	C_19_H_16_N_2_O_4_S	55	243	368.41	61.94	4.38	7.60
							61.90	4.36	7.66
6	2-F	H	C_19_H_14_ClFN_2_O_3_S	52	186	370.40	56.37	3.49	6.92
							56.33	3.44	6.88
6	2-F	H	C_19_H_14_ClFN_2_O_3_S	52	186	370.40	56.37	3.49	6.92
							56.33	3.44	6.88
7	3-OH	3-OH	C_19_H_16_N_2_O_5_S	58	129	348.41	59.37	4.20	7.29
							59.32	4.16	7.24
8	H	Cl	C_19_H_15_ClN_2_O_3_S	55	183	386.85	58.99	3.91	7.24
							58.55	3.89	7.26
9	2,4-CH_3_	4-CH_3_	C_22_H_22_N_2_O_3_S	62	284	394.49	66.98	5.62	7.10
							66.95	5.56	7.05
10	2,5-CH_3_	2-OH	C_21_H_20_N_2_O_4_S	64	302	396.46	63.62	5.08	7.07
							63.58	5.11	7.10
11	2,4-CH_3_	2-OH	C_21_H_20_N_2_O_4_S	58	189	396.46	63.62	5.08	7.07
							63.58	5.11	7.11
12	3,5-CH_3_	2-OH	C_21_H_20_N_2_O_4_S	56	293	396.46	63.62	5.08	7.07
							63.59	5.06	7.02
13	2,4-OH	2-OH	C_19_H_16_N_2_O_6_S	52	243	400.41	56.99	4.03	7.00
							56.95	4.00	7.03
14	H	2-Cl, 4-CH_3_	C_20_H_17_ClN_2_O_3_S	58	109	400.88	59.92	4.27	6.99
							59.88	4.24	6.95
15	H	4-Cl, 3-CH_3_	C_20_H_17_ClN_2_O_3_S	56	113	400.88	59.92	4.27	6.99
							59.88	4.24	6.95
16	2-Cl	4-CH_3_	C_20_H_17_ClN_2_O_3_S	68	279	400.88	59.92	4.27	6.99
							59.95	4.24	6.96
17	2,3,4-OH	4-CH_3_	C_20_H_18_N_2_O_6_S	44	253	414.43	57.96	4.38	6.76
							59.95	4.42	6.72
18	3-OH	2-Cl,5-CH_3_	C_20_H_17_ClN_2_O_4_S	66	173	416.88	57.62	4.11	6.72
							57.59	4.08	6.70
19	2-F	2-Cl,4-CH_3_	C_20_H_16_ClFN_2_O_3_S	60	175	418.87	57.35	3.85	6.69
							57.30	3.81	6.66
20	3,5-CH_3_	2-Cl,4-CH_3_	C_22_H_21_ClN_2_O_3_S	55	308	428.93	61.60	4.93	6.53
							61.56	4.88	6.49
21	2-Cl	4-NO_2_	C_19_H_14_ClN_3_O_5_S	58	282	431.85	52.84	3.27	9.73
							52.86	3.21	9.69
22	2-Cl	2-NO_2_	C_19_H_14_ClN_3_O_5_S	66	174	431.85	52.84	3.27	9.73
							52.86	3.21	9.69
23	4-Cl	2-NO_2_	C_19_H_14_ClN_3_O_5_S	52	240	431.85	52.84	3.27	9.73
							52.86	3.22	9.70
24	2,3,4-OH	2-SH	C_19_H_16_N_2_O_6_S_2_	53	119	432.47	52.77	3.73	6.48
							52.74	3.68	6.44
25	2,4-OH	3-Cl,2-CH_3_	C_20_H_17_ClN_2_O_5_S	50	78	432.88	55.49	3.96	6.47
							55.45	3.94	6.42
26	4-Cl	4-Cl,3-CH_3_	C_20_H_16_Cl_2_N_2_O_3_S	70	302	435.32	46.62	3.13	5.44
							46.58	3.08	5.40
27	4-Cl	2-Cl,5-CH_3_	C_20_H_16_Cl_2_N_2_O_3_S	65	158	435.32	46.62	3.13	5.44
							46.58	3.08	5.40
28	2-Br	2-OH	C_19_H_15_BrN_2_O_4_S	52	113	447.30	51.02	3.38	6.26
							51.10	3.40	6.30
29	2-Br	3-OH	C_19_H_15_BrN_2_O_4_S	58	237	447.30	51.02	3.38	6.26
							51.10	3.40	6.30
30	2-Br	2-NO_2_	C_19_H_14_BrN_3_O_5_S	62	297	476.66	47.91	2.96	8.82
							47.88	2.94	8.85
31	3-Br	4-Cl,3-CH_3_	C_20_H_16_BrCl_2_N_2_O_3_S	67	309	479.77	50.07	3.36	5.84
							50.11	3.31	5.80

### Biological activity

All the synthesized compounds were also evaluated for *in vitro* antibacterial activity against Gram-positive bacteria: *Staphylococcus aureus* (NCIM 2122), *Bacillus subtilis* (MTCC 121), Gram-negative bacteria: *Escherichia coli* (MTCC118), *Pseudomonas aeruginosa* (MTCC 647), *Salmonella typhi* (NCIM 2501), *Klebsiella pneumonia *(MTCC 3384) and fungus *Candida albicans* (MTCC 227), *Aspergillus niger* (NCIM 1056), by using the twofold serial dilution technique and the results are summarized in Table 2. Ciprofloxacin was used as the standard for antibacterial activity and fluconazole was used as the standard for antifungal activity.

**Table 2:** **In** ***vitro*** **antibacterial and antifungal activity data of test compounds: 4-31**

**Minimum inhibitory concentration MIC (μg/mL)**								
**Comp. code**	***S a***	***B.s***	***E.c***	***P.a***	***S.t***	***K.P***	***C.a***	***A.n***
4	50	25	6.25	50	50	50	3.12	6.25
5	50	6.25	25	25	50	50	12.5	25
6	100	50	50	100	100	50	25	50
7	25	50	50	50	25	6.25	6.25	12.5
8	12.5	25	25	50	25	50	12.5	6.25
9	100	100	100	100	50	100	50	50
10	3.12	6.25	6.25	6.25	6.25	6.25	12.5	25
11	25	50	100	100	25	100	12.5	25
12	50	25	25	50	50	25	25	50
13	25	25	50	25	50	50	50	25
14	50	50	25	50	50	100	25	25
15	25	50	12.5	50	50	100	12.5	25
16	100	50	100	100	100	100	>100	>100
17	50	12.5	12.5	50	12.5	25	50	50
18	50	100	50	100	100	100	12.5	25
19	25	50	25	100	50	25	12.5	12.5
20	>100	>100	>100	>100	>100	>100	>100	>100
21	50	25	50	50	50	25	6.25	12.5
22	50	50	50	50	50	50	25	25
23	50	50	50	50	50	50	50	25
24	100	100	6.25	100	100	12.5	12.5	12.5
25	100	100	50	100	50	50	50	50
26	50	50	25	50	50	50	25	25
27	25	25	12.5	50	25	50	6.25	12.5
28	50	50	50	100	50	100	12.5	25
29	25	25	25	100	25	100	25	12.5
30	50	100	50	100	100	>100	25	12.5
31	>100	>100	>100	>100	>100	>100	>100	>100
*CIP.	0.78	0.78	0.78	0.78	0.78	0.78	-	-
*FLU.	-	-	-	-	-	-	12.5	12.5

*S.a: Staphylococcus aureus*, B.s:*Bacillus subtilis*, E.c:*Escherichia coli*, P.a: *Pseudomonas aeruginosa*, S.t: *Salmonella typhi*, K.p: *Klebsiella pneumonia,* C.a: *Candida albicans*, A.n: *Aspergillus niger*.

*CIP: Ciprofloxacin, *FLU: Fluconazole.

Experiments in duplicates.

Compound **10** showed highest activity against all the bacterial strains. Compound **4** and **10** showed highest activity against *E. coli* while compound **24** exhibited moderate activity against the same bacterial strain while exhibited weak activity against all the other bacterial strains. Compound **5** and **7** exhibited highest activity against *B. subtilis and K. pneumoniae* respectively while compound **17** exhibited moderate activity against all the three bacterial strains *B. subtilis, E. coli* and *S. typhi.* Compound **8** exhibited moderate activity against *S. aureus.* However, all these compounds exhibited activity less than that of standard drug ciprofloxacin. Rest of the compounds showed mild to moderate activity against all other bacterial strains.

Compound **4** exhibited excellent activity against the fungal strains *C. albicans* and *A. niger*. In fact, the activity was higher than that of standard fluconazole. Compound **7, 8, 15, 19, 21, 24** and **27** also exhibited very high activity against both the fungal strains comparable to the standard drug. Compounds **5, 10, 11, 18** and **28** exhibited significant activity against *C. albicans* while showing moderate activity against *A. niger.* All other compounds showed moderate to weak antifungal activity against both the fungal strains.

The introduction of phenyl groups substituted at different positions with electronegative groups such as chloro, hydroxyl and nitro enhances the antimicrobial activity, particularly evident in case of antifungal activity. However, the introduction of one methyl group does not have much influence in the increase or decrease of antifungal activity as in case of compounds **19** and **27**. The introduction of two methyl groups in the phenyl ring directly substituted at the 5th position of the thiazolidine −2,4-dione nucleus results in slight decrease in the antifungal activity but increases antibacterial activity as is evident from the activity of compound **10**.

The newly synthesized compounds were evaluated for their HIV-1 RT inhibitory activity. Percentage of inhibition has given in Table 3. Among the synthesized compounds, compound **24** showed significant HIV-1 RT inhibitory activity with 73% of inhibition with an IC_50_ value of 1.31 μM. Compound **23** also showed 58% inhibition, however its IC_50_ value was negligent. Rest of compounds showed weak activity.

**Table 3:** **HIV-RT inhibitory activity**

**Comp. code**	**% inhibition**
4	26.2
5	15.7
6	42
7	17.7
8	31
9	27
10	13.8
11	25.8
12	23.5
13	31.8
14	16.2
15	26
16	11
17	23
18	34
19	41.2
20	18.8
21	34
22	24
23	58
24*	73
25	34
26	23
27	34
28	6.5
29	24.5
30	14.1
31	10
Efavirenz*	98

*IC_50_ (μM) of compound 24 is1.31 & efavirenz* 0.0717, concentration used was 1 μM, experiments in duplicates.

From the results of HIV-1-RT inhibitory activity, it is evident that compound **24** with hydroxyl groups substituted at 2, 3 and 4 positions of the phenyl ring attached at the 5^th^ position of the thiazolidinedione ring with a linker group CH_2_CH_2_CONH at the N-3 position linked to a second phenyl ring substituted with a 2-mercapto group positively influenced the activity.

According to the above obtained data, we found that compounds **4**–**31** exhibited promising antimicrobial activities. In case of HIV-1-RT inhibitory activity, only compound **24** was found to be significantly active while the others exhibited weak non-nucleoside reverse transcriptase activity.

### Binding mode analysis

With the aim of rationalizing the biological data obtained and considering the best obtained *in vitro* results for the compounds **4**–**31,** a molecular modeling study was carried out in order to investigate the possible interactions of the highest active compounds **24**, **10** and **4** with the non nucleoside inhibitory binding pocket(NNIBP) of RT, active site of GlcN-6-P synthase and cytochrome P450 14-α-sterol demethylase from *Candida albicans* (*Candida* P450DM) as the target receptors respectively using the Extra Precision (XP) mode of Glide software [36].

**Figure 2:**
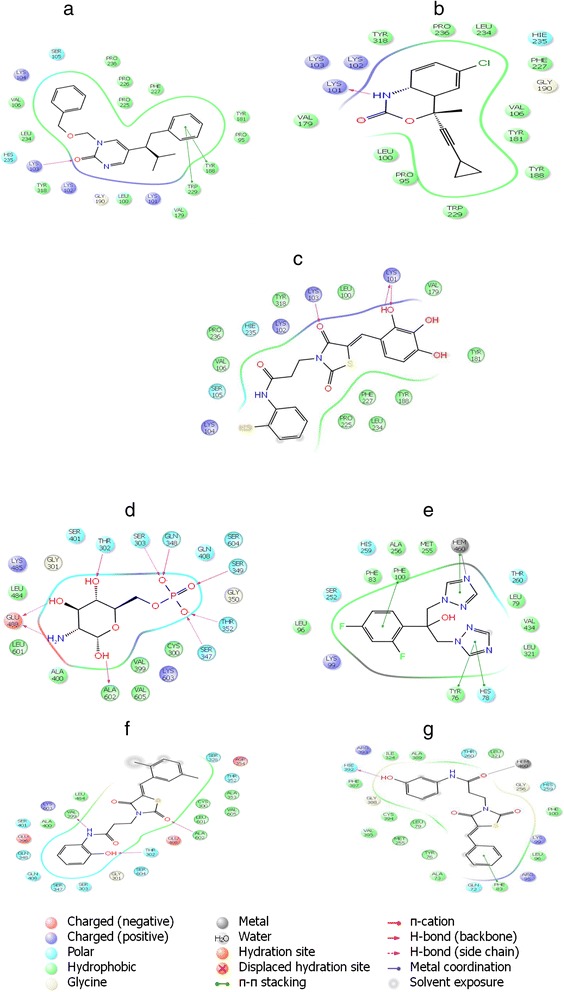
**2D sketch views.** Binding mode of **a)** Ref. ligand (TNK-651) **b)** efavirenz **c)** compound 24 into the NNIBP of 1RT2 **d)** Glucosamine-6-Phosphate (2VF5) **e)** Ref ligand fluconazole (chimeric 1EA1) and compounds **f) 10** in the active site of 2VF5 **g) 4** in the active site of chimeric 1EA1.

To validate the Glide software, firstly the interaction between TNK651 and HIV-1 RT was modeled. Superimposition of the experimental bound (co-crystallized) conformation of TNK651 [41] and that predicted by Glide are shown in Figure 2a. Glide successfully reproduced the experimental binding conformations of TNK 651 in the NNRTI-binding pocket of HIV-1 RT with an acceptable root-mean-square deviation (RMSD) of 2.4 Å. Visual inspection was then performed on the resulting docking solutions of the compound **24** to analyze the binding mode and key protein ligand interactions and was compared with that of the experimentally determined binding mode and interactions of the bound ligand TNK-651 and the standard efavirenz. The key interactions were mainly hydrogen bonding interactions with Lys103 and Lys101 respectively. The carbonyl oxygen at position 4 in the thiazolidinedione moiety forms a strong H-bond interaction with the NH terminal group of Lys103.Another strong H-bond interaction of the hydroxyl group at the ortho position of the first phenyl ring with one of carbonyl oxygen atoms of Lys101 was observed. The phenyl ring in the 2, 3, 4-trihydroxybenzaldehyde moiety along with the thiazolidinedione moiety was oriented in the bigger hydrophobic pocket formed by Phe227, Pro225 Leu234 Tyr181, Tyr188, Leu100 and Val179 while the CH_2_CH_2_CONH linker showed favorable interaction with the amino acid residues Tyr318, Pro236 and Val106. Docking score of the compound **24** (Glide XP score-11.30) was lower than the bound ligand TNK-651(Glide XP score-13.29) but comparable to that of standard efavirenz (Glide XP score-11.33) .Possible interactions for the reference ligand TNK-651, efavirenz and compound **24** have been shown in Figure 2a,b and c respectively.

The active site of GlcN-6-P synthase (PDB code 2VF5) consists of 16 amino acid residues as Glu488, Ser303, Ala602 Ser347, Ser349, Gln348, Thr302, Thr352, Val605, Ala400, Cys300, Val399, Leu601, Leu484, Ser401 and Lys603 as shown in Figure 2d. Figure 2d and f shows the docked poses of the reference ligand and of highest active compound **10** respectively. The binding mode of GlcN-6-P synthase (2VF5) with its bound inhibitor glucosamine-6-Phosphate (Figure 2d) shows 9 hydrogen bonds with residues Glu488, Ser303, Ala602, Ser347, Ser349, Gln384, Thr302, Thr352, hydrophobic interactions with Val605, Ala400, Cys300, Val399, Leu601, Leu484 and electrostatic interactions with Ser401 and Lys603 with a docking score −7.16. Docking validation was performed with an RMSD value of 1.674 Å ensuring precision and reproducibility of the docking process. The docked pose of active compound **10** (Glide XP score −4.89) has been depicted in Figure 2f. It was interesting to note that three important hydrogen bonds are formed by compound **10.** The hydroxyl group on the benzylidene moiety forms a hydrogen bond with the carbonyl oxygen of Thr302. The NH in the CH_2_CH_2_CONH linker forms a strong hydrogen bond with the carbonyl oxygen of Val399. Another strong H-bond is evident between the carbonyl oxygen of thiazolidinedione with the NH terminal group of Ala602. The 2-hydroxyphenyl group makes favorable interaction with the side chain of Ala 400. The thiazolidinedione ring shows favorably oriented towards the residues Leu601, Cys300, Val605 and Ala353. The linker (CH_2_CH_2_CONH) shows favorable interactions with Leu484.

As the target enzyme *Candida* P450DM is a membrane-bound enzyme, it is difficult to crystallize by X-ray analysis; therefore, no experimental data has been available for the structure of this enzyme. However, the crystallographic structure of the complex between cytochrome P450 14-α-sterol demethylase from *Mycobacterium tuberculosis* (*Mycobacterium* P450DM) and fluconazole is present in the Protein Data Bank with the ID 1EA1. A perusal of the literature showed that high homology exists between the two analogous enzymes, *Candida* P450DM and *Mycobacterium* P450DM [42].

A chimeric enzyme of *Candida* P450DM complexed with fluconazole was modeled following the procedure of Rosello *et al.* [42]. Fluconazole maintained practically the same orientation as in 1EA1 with a docking score of −6.01. In fluconazole, interaction of triazole ring with heme is coordination of N atom with Fe atom of heme, while another triazole ring also forms π– π stacking interactions with Tyr78 and His78. The diflurophenyl group also forms π–π stacking interactions with Phe100. Phenyl ring shows hydrophobic interactions with amino acid residues Leu79, Leu96, Phe83, Met255, Ala256, Leu321 and Val 434. Docking validation was performed with RMSD value of 2.094 Å ensuring precision and reproducibility of the docking process (Figure 2e).

To illustrate the binding mode of the newly synthesized compounds in the active site of chimeric 1EA1, the docked pose of the highest active compound **4** (Glide XP score −8.13) has been analysed as follows (Figure 2g). The key interactions are mainly hydrogen bonding interactions. The hydroxyl group on the benzylidene moiety forms a hydrogen bond with Hie392. The CO in the CH_2_CH_2_CONH linker forms a strong coordinate bond with Hem460. The aromatic ring forms π–π stacking interactions with Phe83 residue. The thiazolidinedione ring is oriented in the hydrophobic pocket formed by Tyr181, Phe100, Met255, Val395 and Leu79. Both phenyl rings attached to the thiazolidinedione nucleus makes favorable interactions with the side chains of Leu96, Al173, Tyr76, Cys394, Phe387, Ile324, Ala389 and Leu321.

These docking results demonstrate that hydrogen bond interactions, hydrophobic interactions and the coordinate bond with the Hem residue in 1EA1 are very important for binding of compound **4** with the active site residues and may be responsible for the very high antifungal activity as shown by compound **4.**

## Conclusion

In the present study, a series of thiazolidinedione analogs have been synthesized and their structures have been characterized by IR, NMR and mass spectroscopy. All the newly synthesized compounds were tested for HIV-1- RT inhibitory activity by microplate assay method and for antimicrobial activity by two fold serial dilution method. From the modeling studies as well as from the SAR, electronegative groups substituted at various positions of the phenyl rings with a thiazolidinedione scaffold may be responsible for the very high HIV-1-RT inhibitory activity of compound 24. In case of antibacterial activity the methyl groups substituted in the phenyl group attached to the 5^th^ position of the thiazolidinedione ring system plays a significant role while in case of antifungal activity the electronegative groups, particularly hydroxyl groups substituted in the various positions of both the phenyl groups are responsible for enhanced antifungal activity. The study encourages us to consider a new molecular skeleton of thiazolidinediones substituted at the 3^rd^ and 5^th^ position by aryl groups with adequate spacers may be identified as a potential lead compound for the development of ant-HIV agents with the ability to combat opportunistic bacterial and fungal infections.

## Abbreviations

Å: Angstrom
AIDS: Acquired Immuno Deficiency Syndrome
AR: Analytical reagent
°C: Degree centigrade
DMSO: Dimethyl sulfoxide
CD4: Cluster of differentiation 4
CFU: Colony forming unit
GHz: Giga hertz
GR: Guaranteed reagent
h: Hour
Hz: Hertz
HIV: Human Immuno Deficiency Virus
HIV-1: Human Immuno Deficiency Virus Type-1
^1^H NMR: Proton Nuclear Magnetic Resonance
IR: Infrared
IC_50_: 50% Inhibitory Concentration
MIC: Minimum Inhibitory Concentration
MS: Mass Spectroscopy
MTCC: Microbial Type Culture Collection
NCIM: National Collection of Industrial Microorganisms
NNRTIs: Non Nucleoside Reverse Transcriptase Inhibitors
OPLS: Optimized potentials for liquid simulations
PDB: Protein Data Bank
RMSD: Root mean square deviation
RT: Reverse transcriptase
SAR: Structure activity relationship
XP: Extra precision
